# Impact of Simulation Training on Undergraduate Clinical Decision-making in Emergencies: A Non-blinded, Single-centre, Randomised Pilot Study

**DOI:** 10.7759/cureus.7650

**Published:** 2020-04-12

**Authors:** James Everson, Alice Gao, Carrie Roder, John Kinnear

**Affiliations:** 1 Emergency Medicine, School of Medicine, Anglia Ruskin University and Southend University Hospital, Chelmsford, GBR; 2 Emergency Medicine, School of Medicine, Anglia Ruskin University, Chelmsford, GBR; 3 Medical Education and Simulation, School of Medicine, Anglia Ruskin University, Chelmsford, GBR; 4 Internal Medicine: Critical Care, School of Medicine, Anglia Ruskin University, Chelmsford, GBR

**Keywords:** undergraduate medical education, simulation, decision making, e-learning, emergency medicine

## Abstract

Introduction

There is an increasing evidence base for the use of simulation-based medical education. Simulation is superior to more didactic methods for teaching a range of technical and non-technical skills, and students report they often derive more educational value from it compared with other teaching methods. There is currently limited evidence that simulation training improves clinical decision-making and, therefore, this pilot study sought to explore this further.

Methods

Students were randomised to take part in either five classroom tutorials and simulation training sessions or five classroom tutorials and an online e-learning module. On completion of the teaching, students all undertook an unseen assessment scenario (managing a simulated patient with anaphylaxis), where they were scored using a weighted marking scheme. The time taken to make decisions and student-reported confidence in decisions were also measured.

Results

14/14 simulation-group participants and 12/14 e-learning-group participants completed the post-learning assessment. The simulation group identified anaphylaxis and gave adrenaline more quickly (p 0.008 and 0.005, respectively), and this cohort was more confident in making the diagnosis (p 0.044). There was no statistically significant difference between weighted global assessment scores for each group (p 0.705). The e-learning group called for help more quickly (p.0.049), although fewer students in this group called for help (five vs. nine). There was no statistical difference in confidence in decisions to administer adrenaline or call for help (p 0.539 and 0.364 respectively).

Conclusions

Participants who undertook simulation training were able to more confidently and quickly identify the diagnosis and initiate emergency treatment. However, there was not a statistically significant difference between groups using an overall weighted score. Using simulation to train students to perform better in emergencies and improve their decision-making shows promise but a further quantitative study is required.

## Introduction

Medical students in the United Kingdom (UK) often feel under-prepared for entering clinical practice as a foundation doctor, often citing a lack of practical clinical experience and training in managing uncertainty as reasons for this. Particular domains in which they lack confidence include managing emergency situations and clinical decision-making [1-3]. The use of simulation training shows promise for helping to improve these skills in medical undergraduates, as it provides learners with the opportunity to experience realistic clinical scenarios, to make decisions, and face uncertainty [4]. Simulation training is increasingly being incorporated into undergraduate curricula, and there is a growing evidence base that it is beneficial for learners [5]. The United Kingdom General Medical Council, in its standards for medical education and training, states that “Learners must have access to technology enhanced and simulation-based learning opportunities within their training programme…” [6]. Some of the key mechanisms by which simulation is thought to support effective learning and, in particular, the development of prioritisation and decision-making skills, include repetitive practice, provision of personalised feedback, and the option of varying difficulty [7]. Improved clinical decision-making is arguably a more patient-centred outcome than considering improved student confidence, which has been more extensively studied, although it is more complex to objectively measure the efficacy of decision-making or prioritisation.

There is a well-developed evidence base, particularly from post-graduate studies, that simulation training is superior to traditional educational methods (such as lectures) for training specific procedural skills such as laparoscopic surgical procedures [5,8-9]. Additionally, medical students consistently report that they find simulation training to be educationally valuable, and it improves their confidence in challenging and uncertain situations [10-11]. This specifically includes improving confidence in making decisions in emergency situations [12-13].

At present, however, there is limited evidence that simulation training actually leads to better clinical decision-making in emergency situations, and there is not necessarily a consensus on what comprises ‘better’ decision-making. Studies have demonstrated improved performance by undergraduate medical students in Objective Structured Clinical Examination (OSCE) assessments and quicker and more accurate decisions by resuscitation team leaders in a simulated setting following simulation training [14-15]. The present study sought to build on these findings, by using a global weighted marking scheme to attempt to quantify students’ overall decision-making and by comparing simulation training to an online learning (e-learning) package. The use of e-learning as a comparison was selected, as this modality is now frequently being used in undergraduate medical education, with some of its advantages reported to be that it can deliver a standardised experience to every student while being flexible in terms of how and when it is engaged with [16]. Moreover, the use of e-learning instead of classroom-based teaching potentially represents a cost-saving of up to 50%. As such a finding that e-learning is non-inferior to simulation training could be particularly significant for those involved in curriculum design and budget management [17].

Objective

This study aimed to determine whether undergraduate medical students participating in simulation and classroom tutorial teaching make clinical decisions that are quicker, more accurate, and in which they are more confident than students who receive classroom teaching and an e-learning module.

## Materials and methods

Third-year undergraduate medical students from a UK medical school were invited to participate in the study at one hospital site. All students within this group were eligible to participate. Recruitment ran from September 2018 until March 2019. The sample was convenience-based, as the researchers were already working at the study hospital site and used an established teaching programme as part of the research. All students reported they had limited simulation exposure (less than five sessions) and training on the 'Airway, Breathing, Circulation, Disability, Exposure' (ABCDE) approach to assessing and treating an unwell patient at the time of enrolment [18]. Twenty-eight (28) students volunteered and were randomised to take part in either five weekly classroom tutorials and simulation training sessions or the same classroom tutorials with five online e-learning sessions. In two cohorts of 14 students, each student was assigned a number between one and 14 in order of enrolment. These numbers were then drawn from a hat and alternately allocated to each study group to ensure equal group sizes for the teaching sessions.

For the simulation group students, sessions were delivered as a one-hour classroom tutorial followed by a one-hour simulation session, during which each student led on a simulated scenario assessing and managing an unwell patient. In the e-learning group, students completed the same one-hour classroom tutorials (separately from the other group) and then completed the associated e-learning (roughly one hour of material provided for each session) in their own time each week, with all students self-reporting completion of the e-learning. Over the five weeks, students covered a different letter of the ABCDE approach each week (‘Airway’ on week one, ‘Breathing’ on week two, and so on), ‘Disability’ and ‘Exposure’ were covered together on week four, and week five was a summary session, incorporating all aspects of the ABCDE approach. Each week involved the consolidation of learning and concepts from the prior week(s).

Two separate cohorts of 14 students, with seven students in each group, were recruited. Ethical approval for this study was received through both the Anglia Ruskin University research ethics committee and local hospital research and development department.

The teaching on the ABCDE approach to assessing and treating an acutely unwell patient was based upon guidance from the UK Resuscitation Council (RCUK). Students were taught by the same tutors (who had formal training in both simulation and education) and using identical lesson plans, although they were taught separately. The e-learning materials were designed by the same tutors and were a collection of single best-answer questions with detailed feedback.

Once students had completed all the teaching, they individually participated in a seven-minute-long, unseen simulated assessment scenario, with two assessors present. All students completed the same scenario, which was based upon the assessment and treatment of a patient with anaphylaxis. Anaphylaxis was chosen as the assessment topic, as there are both specific signs and symptoms of this condition and specific treatments that must be given that represent an effective response. The essential treatments include the administration of intramuscular adrenaline, steroids, chlorphenamine, intravenous fluids, and supplemental oxygen. Assessors followed a structured script and the scenario followed a fixed progression; see Table 1 below.

**Table 1:** The script used by assessors in the simulated assessment scenario that study participants completed following the educational interventions It highlights key verbal cues, observations, and findings for each aspect of the clinical examination. The patient and associated details are fictitious and any resemblance to a person living or deceased is a coincidence. GCS: Glasgow Coma Scale; ECG: electrocardiography

Assessment scenario script
Candidate brief: You are called to assess 26-year-old George, who is complaining of feeling generally unwell, with an itchy chest. He has just had his lunchtime dose of co-amoxiclav for community-acquired pneumonia.
Initial observations (when the candidate asks):
Oxygen saturation: 94% on air (98% on oxygen)
Respiratory rate: 22
Heart rate: 103
Blood pressure: 112/67
Temperature: 36.7
Examination findings:
A - Patent, talking & orientated. Complaining of itchiness
B - Chest clear. Normal expansion/percussion
C - Warm peripheries, capillary refill <2s. Normal heart sounds. ECG shows sinus tachycardia
D - GCS 15/15, glucose 5.7, pupils equally reactive to light
E - Urticarial rash across torso. No other findings
At 3 minutes or end of initial ‘exposure’ assessment situation changes
“George is complaining of feeling breathless and very unwell”
Repeat observations (when the candidate asks):
Oxygen saturation: 88% on air (94% if on oxygen)
Respiratory rate: 36
Heart rate: 124
Blood pressure: 87/48 (improves if adrenaline and/or fluid challenge given)
Temperature: 36.4
Repeat examination findings:
A - Stridor. Swelling of lips and tongue. Remains patent
B - Diffuse wheeze. Tachypnoeic. Normal percussion/expansion
C - Cool peripheries. Capillary refill 4s peripherally, 4s centrally. Normal heart sounds. ECG: sinus tachycardia
D - GCS 15/15, glucose 5.6, pupils equally reactive to light
E - Diffuse urticarial rash across torso/trunk/thighs
Scenario ends at 7 minutes

A weighted marking scheme (Table 2) was designed using a Modified Delphi approach [19].

**Table 2:** The weighted marking scheme used to score study participants on the different decisions made during the simulated assessment scenario The weighting for each domain was agreed by expert consensus. Bold points reflect ‘key’ decisions and non-bold points can only be scored if the preceding, overarching bold decision has been made. Points were only given for hydrocortisone or chlorphenamine if students gave these at the correct dose and/or route, hence the dash next to these bold points.

Decision	Weighted score
Oxygen	2
Correct O_2_ flow rate	1
Correct O_2_ device	1
IV access	1
Appropriate site	1
Appropriate size	1
Identify anaphylaxis	3
Administer adrenaline	5
Correct adrenaline dose + route	3
Unsafe dose/route	-3
IV fluids (appropriate)	3
Administer hydrocortisone	-
Correct dose	1
Correct route	1
Administer chlorphenamine	-
Correct dose	1
Correct route	1
Call for help	5
Medical emergency team called	2
TOTAL	32 (-3)

**Figure 1:**
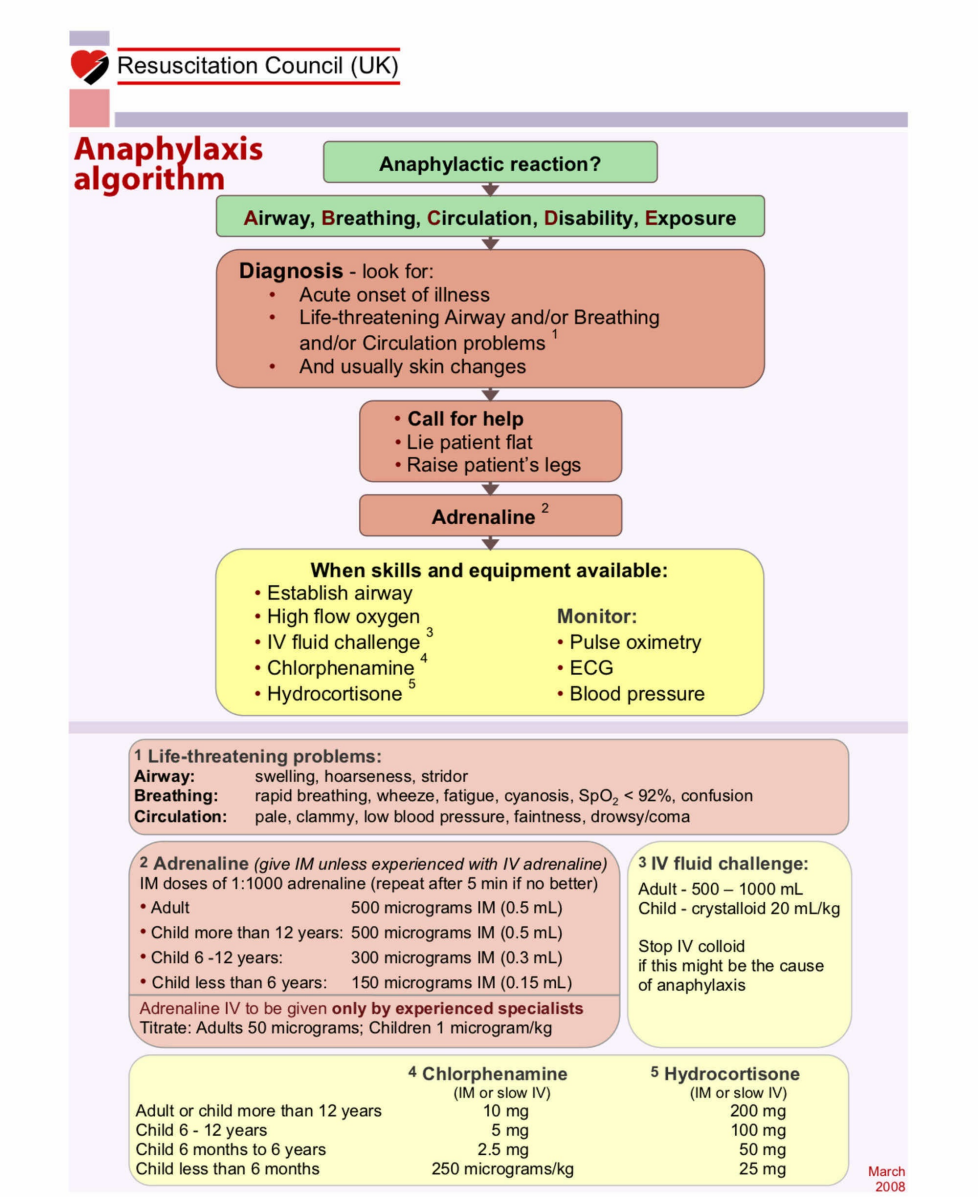
The Resuscitation Council (UK) algorithm for managing anaphylaxis. Weighting for the marking scheme was based upon the order in which interventions are prioritised in this algorithm. Source: Reference [20]

The marking scheme was based upon the Resuscitation Council (UK) (RCUK) anaphylaxis algorithm (Figure 1).

During the scenario, the timing of key student decisions was recorded, and immediately on completion of the scenario, students were asked to rate their confidence on a 10-point scale in various decisions they made, with their confidence rating reflecting an overall impression of how timely decisions were made and how confident they were with the doses and routes of medications, for example.

## Results

Twenty-six (26) out of 28 students completed the assessment scenario: 14/14 in the simulation group and 12/14 in the e-learning group (two missed the assessment due to sickness). The primary outcome measure was the difference in the total weighted score for the assessment. These data were not normally distributed so an independent samples Mann-Whitney U test was used for analysis. The simulation group (n = 14) had a mean score of 20.86/32 and the e-learning group (n=12) had a mean score of 19.58/32; there was no significant difference between the two groups, p 0.705.

**Figure 2:**
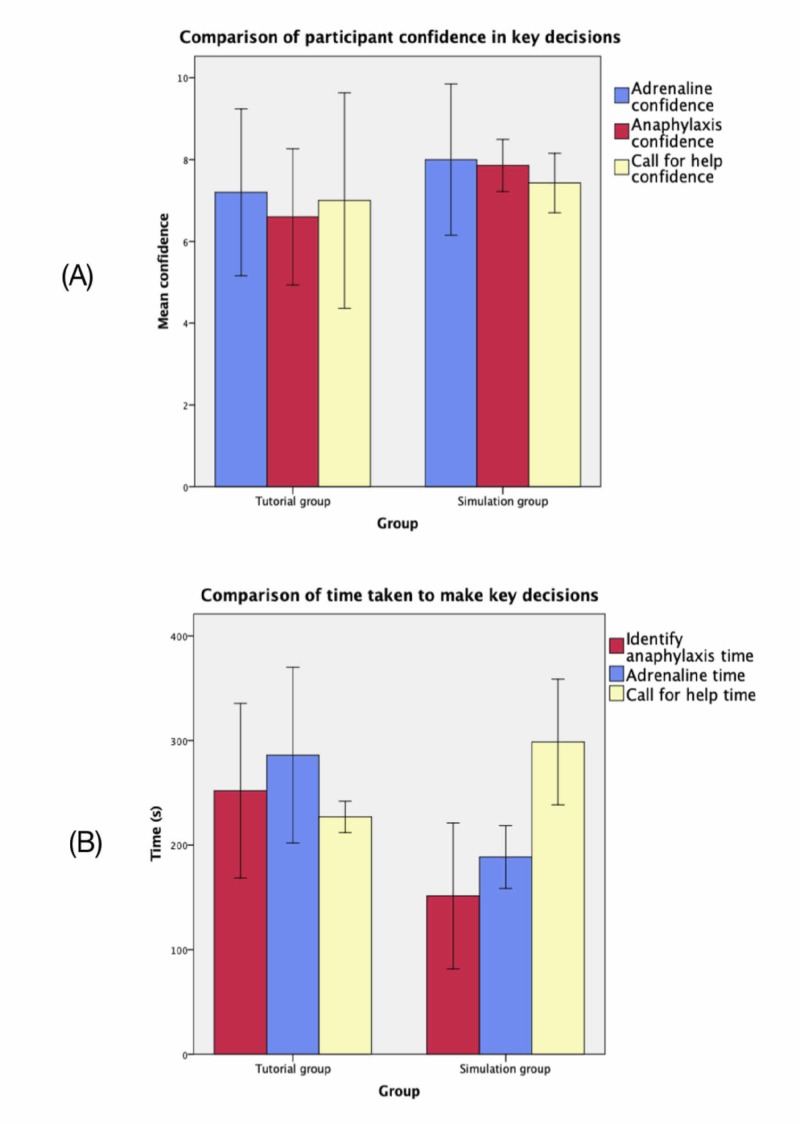
Graph (A) represents the mean confidence (scored /10) reported by participants in the three key outcome decisions and is separated by intervention and control group. Graph (B) represents the average time for the three key outcome decisions and is separated by intervention (simulation) and control (e-learning) group The error bars on both graphs represent the 95% confidence interval.

Secondary outcome measures included time to administer adrenaline, time to identify anaphylaxis, time to call for help, and participant subjective confidence in these decisions. Table 3 shows the primary and secondary outcome measure results. An independent samples Mann-Whitney U test was used to analyse non-parametric data, while an independent samples t-test was used for parametric data. Figure 2 also graphically represents the key outcome measures of time to identify anaphylaxis, administer adrenaline, and call for help for both groups.

**Table 3:** Depiction of primary and secondary outcome measures, with mean recorded values for each research group and the number of participants included for each measurement A statistically significant difference between groups is signified by a p-value of <0.05. The 95% confidence interval is also shown.

Outcome measure	Simulation group mean	E-learning group mean	p value	95% Confidence Interval
Total weighted score	20.86/32 N=14	19.58/32 N=12	0.705	N/A
Time to identify anaphylaxis	173.85(s) N=13	243.33(s) N=12	0.008	19.852 to 119.122
Confidence in identifying anaphylaxis	7.54/10 N=13	6.50/10 N=12	0.044	-2.049 to -0.028
Time to give adrenaline	194.58(s) N=12	264.50(s) N=10	0.005	23.209 to 116.624
Confidence in decision to give adrenaline	7.89/10 N=12	7.00/10 N=12	0.539	N/A
Time to call for help	287.78(s) N=9	227.00(s) N=5	0.049	-121.324 to -0.231
Confidence in decision to call for help	7.58/10 N=12	7.30/10 N=12	0.364	N/A

## Discussion

The findings that students who completed simulation training made more rapid decisions, and were more confident in these decisions, are consistent with evidence from previous studies. This supports the idea that simulation training is beneficial for developing a wide range of clinical and non-clinical skills [4-5,8]. There are a number of potential reasons why simulation training leads to better performance, and there is not necessarily a consensus in the current literature although there are recurrent themes. Compared with other educational methods, students often receive more personalised feedback and they frequently benefit from a formal scenario ‘debrief,’ which is immediate and structured feedback directly relevant to the learning event, which further supports learning and development [21]. Furthermore, students participating in the simulation have the opportunity to make mistakes in a safe and supported environment, and through this experiential learning process, may subsequently perform better and make fewer mistakes in similar scenarios in future [21]. There is also likely to be a benefit from the experience of repetitively practising the ABCDE approach to an acutely unwell patient in simulation training, combined with improved familiarity with key resuscitation equipment as a result of this and familiarity with the structure and progression of a simulated (or real) patient assessment. This may mean students are able to focus less on the ‘process' of assessing a simulated patient and are able to devote more attention to making decisions pertaining to the prompt diagnosis and initiation of key investigations and treatments. While students completing classroom tutorials and e-learning are still going through a repetition of relevant theory, they lack exposure to the authentic practice of working in a team, making clinical decisions, and prioritising clinical tasks. This may, in part, account for the improved performance of students who have completed simulation training since supervised practice is likely an important mechanism by which students learn and improve their future practice [7].

The pragmatic use of e-learning for our control group of learners is reflective of the increased use of this teaching modality in medical schools, in place of lectures or other didactic methods that are becoming less popular in modern education [22]. Simulation is costly and requires expensive simulation equipment and facilities, as well as a high instructor to students ratio. As such, if an e-learning package is almost as effective as delivering simulation training, there is a question as to whether, overall, it is the most efficient for educational institutions to use e-learning preferentially.

While the overall weighted score was used as the primary outcome measure, the time to administer adrenaline is arguably an equally important outcome measure, as evidence suggests that in iatrogenic cases of anaphylaxis, the median time to cardiac or respiratory arrest is five minutes, so administering adrenaline more quickly can improve patient outcomes [23]. The time to administer adrenaline may, therefore, better represent a student’s ability to prioritise and make an effective clinical decision in this instance, recognising it is the critical intervention in treating anaphylaxis. The use of a weighted binary mark scheme in this study did not account for the order in which students made decisions nor how quickly they were made. For this reason, the finding that students who had received simulation training made this critical decision significantly more quickly is potentially more important than the finding that there was no difference in overall weighted total scores.

The fact that no significant difference was identified between the groups in the context of the overall weighted score may be due to a number of factors, the most obvious of which is the small sample size. The marking scheme itself was based on expert consensus of what decisions and interventions are most important when managing anaphylaxis, but the relative weighting of each decision remains subjective and open to debate. The advantage of using a marking scheme where points are awarded for completing a given task is that it quantifies performance. Higher scores indicate that students have completed more key interventions (all scoring domains represented ‘key’ decisions only), but it does not reflect the timeliness, nor the order, of those interventions. It could be argued that the timeliness or order of decisions is less important in the context of a brief clinical scenario, and the number of accurate decisions in this time better represents ‘good’ decision-making, but, nonetheless, in a true emergency, such as anaphylaxis, the time to giving adrenaline remains critical.

Strengths and limitations

There are several limitations to this study. There was no baseline testing of students to identify significant differences between candidates pre-intervention and to compare the relative improvement in individual students’ scores following the teaching intervention. To mitigate for the potential differences in prior knowledge, care was taken to ensure all students had been taught about anaphylaxis during the educational component of the study through the tutorials, simulation sessions, and e-learning package. We were unable to control for any separate learning that took place outside of the study. Regarding the marking scheme, students were only scored for making decisions that were verbalised and because students were not prompted or asked clarifying questions, it is possible that students may have missed out on marks due to not verbalising their thoughts.

Undoubtedly, the simulation-based assessment method that was used may have benefitted the simulation group of learners due to familiarity. The method was used primarily for its utility in testing authentic decision-making in the context of an emergency scenario, where non-cognitive skills are important [24]. To mitigate this effect, the simulation-based teaching was distinctly different from the assessment scenario, where students participated in teams rather than as individuals, as was required by the assessment.

Researcher bias was a risk, as the two researchers acted as both tutors during the educational component and assessors in the test scenario. This was mitigated to some degree by the use of a structured marking scheme and a formatted script for the scenario progression. Given the obvious difference in teaching methods used for each study group, it was not possible to blind participants or researchers.

The sample size and use of a single-site with medical students from a single medical school limit the generalisability of the findings, but data from this pilot can inform a power calculation for a larger-scale study in the future.

## Conclusions

There are already established benefits for using simulation training in a variety of contexts, but its specific role in improving decision-making and emergency care skills is not proven. It is likely that simulation training can be as good as, if not superior to, classroom-based teaching or e-learning for training these skills, although further quantitative study on a larger scale is required to provide more conclusive data. It remains to be seen whether the improved confidence students report as a result of simulation training persists after a significant period of time or if they do not participate in regular simulation, as repeated practice is important for the retention of skills and knowledge. The use of weighted decision-making marking schemes needs to be studied further, to identify if they can reliably score the effectiveness of decision-making and differentiate between different groups who have been exposed to different educational methods.
